# Supplementary material for: NetAllergen, a random forest model integrating MHC-II presentation propensity for improved allergenicity prediction
Source: Bioinform Adv. 2023 Oct 16;3(1):vbad151. doi: 10.1093/bioadv/vbad151 (PMC10603389; doi:10.1093/bioadv/vbad151)
Supplement: vbad151_Supplementary_Data [file vbad151_supplementary_data.docx]

# Supplementary material

**Supp. Table 1. Hyperparameters tuning in random forest**

| Hyperparameters | Range |
| --- | --- |
| Number of estimators | 40–200 with a step of 20 |
| Depth of trees | 4–20 with a step of 2 |
| Minimum samples for split | 2, 4, 8 |
| Minimum samples on a leaf | 1, 2, 4 |
| Impurity criteria | gini, entropy |

**Supp. Table 2. The composition of the evaluation dataset**

| Database | Positive data size | Negative data size | Data size |
| --- | --- | --- | --- |
| Allergen Nomenclature | 106 | 487 | 593 |
| AllerBase | 136 | 682 | 818 |
| COMPARE | 27 | 131 | 158 |
| Allergome | 767 | 3,514 | 4281 |
| SDAP | 1 | 5 | 6 |
| Total | 1,037 | 4,819 | 5,856 |

Note: The allergen protein sequences that were identical to allergens already present in the training dataset were excluded. The non-allergen sequences were collected following the same criteria as for the training dataset (detailed in Material and Methods section).

**Supp. Table 3. Cross-validation model performance with incremental feature spaces**

| **Model** | AUC | AUC 0.1 | PPV |
| --- | --- | --- | --- |
| 20F | 0.869 | 0.348 | 0.544 |
| 40F | 0.908 | 0.494 | 0.644 |
| 60F | 0.913 | 0.509 | 0.646 |

Note: The random forest feature spaces were divided into three different subspaces: 20F, 40F and 60F. The 20F model included physicochemical properties, local structural features, and MHC class II presentation propensity. The 40F model included the 20F features + 20 amino acid compositions. The 60F model included 40F + 20 features for evolutionary information for each amino acid (auto-covariance). Detailed information about the feature construction is explained in Material and Methods section.

**Supp. Table 4. Feature compositions in the random forest models**

| Model | MHC presentation propensity (2) | Physicochemical properties (12) | Hydropathy (2) | Secondary structure (4) | Composition (20) | Evolutionary information (20) |
| --- | --- | --- | --- | --- | --- | --- |
| 18F | – | + | + | + | – | – |
| 20F | + | + | + | + | – | – |
| 40F | + | + | + | + | + | – |
| 56F | + | + | + | – | + | + |
| 58F | – | + | + | + | + | + |
| 60F | + | + | + | + | + | + |

Note: The name of the model represents the number of features included in the model. “+” indicates that the features in this category were included in the current model, while “–” indicates non-included features.

1. MHC presentation propensity: binder 1 (HLA-DRB1*04:01, HLA-DQA1*02:01-DQB1*02:02, HLA-DQA1*04:01-DQB1*03:01, HLA-DQA1*01:03-DQB1*06:01, HLA-DQA1*03:01-DQB1*03:02, and HLA-DQA1*05:01-DQB1*02:01) and binder 2 (HLA-DRB1*15:01)

2. Physicochemical properties: average residue weight, average charge, isoelectric point; tiny, small, aliphatic, aromatic, non-polar, polar, charged, basic, and acidic amino acid compositions

3. Hydropathy: hydrophilicity and hydrophobicity

4. Secondary structure: alpha helix, beta sheet, average relevant solvent accessibility

5. Composition: 20 amino acid compositions

6. Evolutionary information: auto covariance of the 20 amino acids

**Supp. Table 5. AlgPred2 validation dataset benchmark**

| Model | AUC | AUC 0.1 | PPV |
| --- | --- | --- | --- |
| RF_60F | 0.886 | 0.551 | 0.712 |
| Baseline | 0.923 | 0.829 | 0.848 |


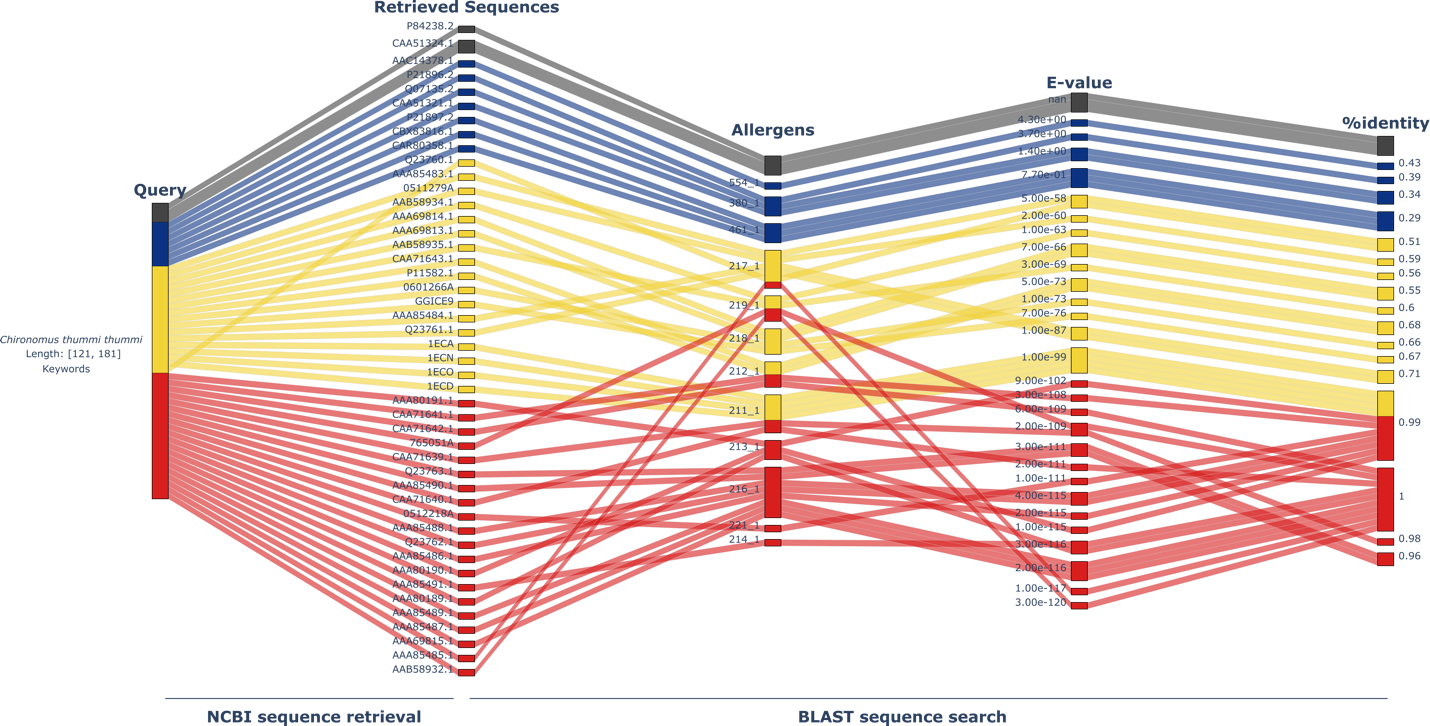


**Supp. Figure 1.** Sequence alignment of non-allergen candidates against the allergen dataset for negative dataset collection. The first column represents the query of species (*Chironomus thummi thummi* in this example) matching the allergen species. Boxes of the second column correspond to putative non-allergens from the same species and applied filters (length and annotation keywords). The putative non-allergens sequences are then compared to the positive allergen dataset. The third column represents the alignments of the putative negative to known allergens by BLAST, and the fourth and fifth columns are E-value and % identity respectively. We observe high similarity to the putative negatives to the allergens. Similarities from high to low were represented by red (≤ 1x10 ^-100^), yellow (1x10-10 ≤ E-value < 1x10-100), and blue (> 1x10-10). Random selection of candidates without filters is likely to have mislabel problems in training. Grey lines indicate no-hits non-allergens in BLAST. Only blue aligned or grey putative non-allergens were selected as negatives.


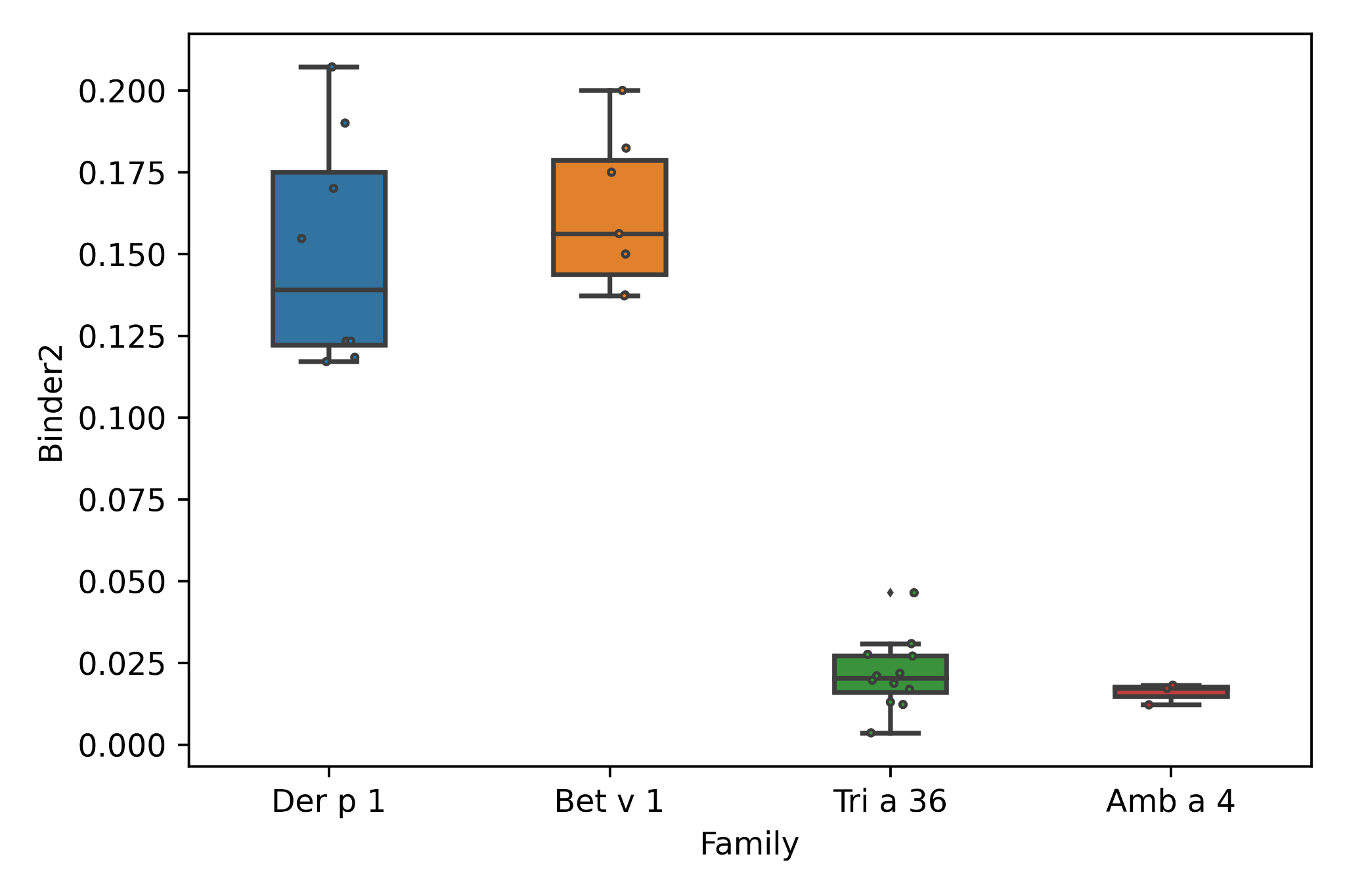


**Supp. Figure 2.** The distribution of binder2 feature (DRB1*15:01) of four types of allergen families. The Der p 1, Bet v 1 families have higher propensity of being presented by DRB1*15:01 MHC molecules, which aligns with previous studies about association between DRB1*15:01 and allergy.

**
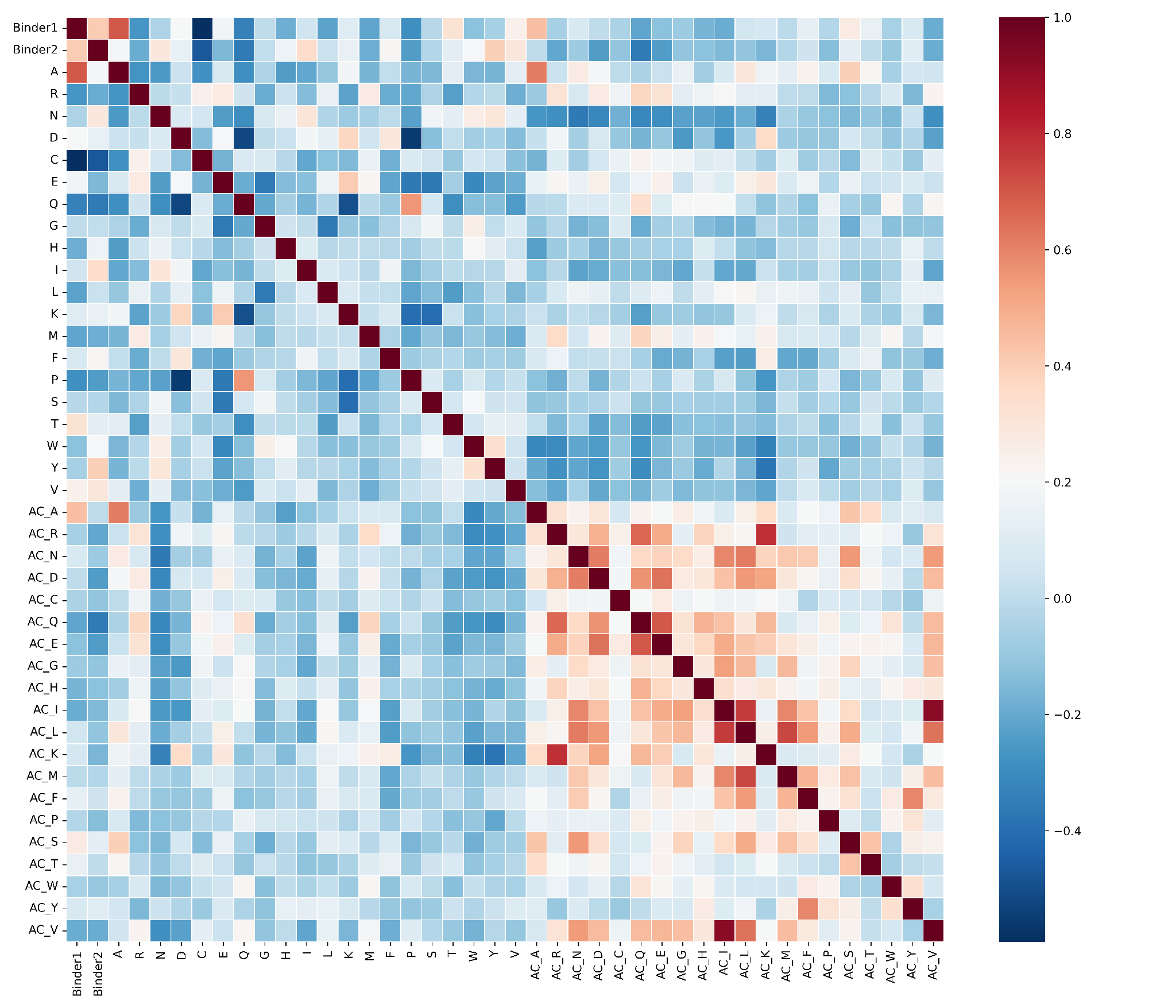
Supp. Figure 3.** Pearson correlation coefficient of MHC II presentation features (binder1, binder2), amino acid compositions (A, R, N, etc.) and autocovariances (AC_A, AC_R, AC_N, etc.) for allergens.


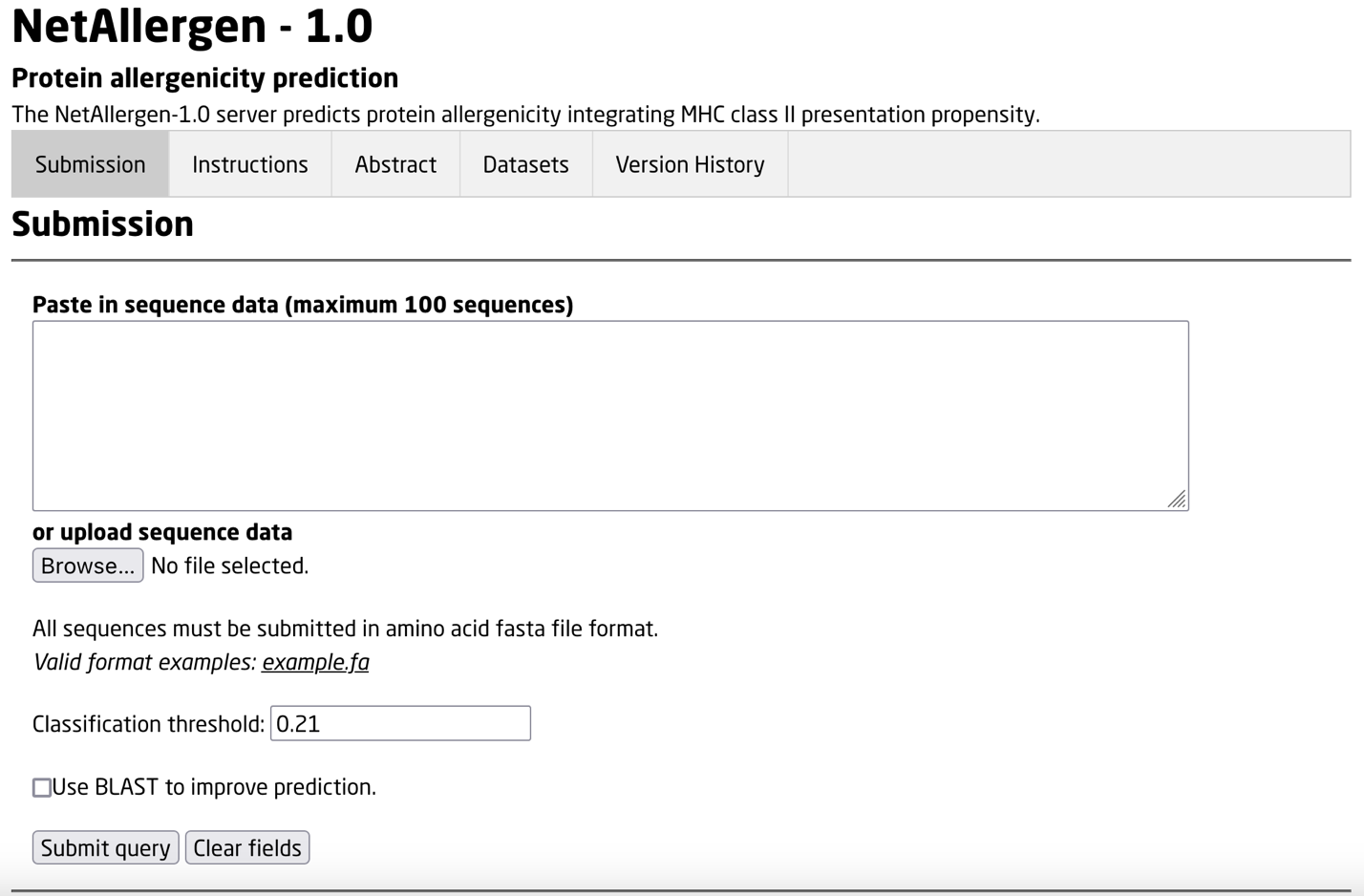


**Supp. Figure 4.** Web service of NetAllergen-1.0.
